# Infection preventive behaviors and its association with perceived threat and perceived social factors during the COVID-19 pandemic in South Korea: 2020 community health survey

**DOI:** 10.1186/s12889-022-13755-z

**Published:** 2022-07-19

**Authors:** Woo In Hyun, Yoon Hee Son, Sun Ok Jung

**Affiliations:** College of Nursing, Ewha Woman’s University, 52 Ewhayeodae-gil, Seodaemun-gu, Seoul, 03760 Republic of Korea

**Keywords:** COVID-19, Preventive behavior, Perceived threat, Perceived social factors, Multiple regression, Urban–rural health behavior

## Abstract

**Background:**

This study examined why some individuals have not properly performed health prevention behavior during the coronavirus disease 2019 (COVID-19) pandemic. We used data from a community health survey conducted by public health centers in South Korea to identify factors affecting COVID-19 prevention behavior in urban and rural areas. Also, we examined whether individual-level demographic, socio-psychological, and structural variables affected COVID-19 prevention behavior by referring to a model explaining individuals’ health prevention behavior. In particular, the study is significant as not many other measures were suggested besides compliance with personal quarantine rules during the early phase of the pandemic in 2020. We hope that the results of this study will be considered in further analysis of infection preventive behavior and in future health crises.

**Methods:**

Probability proportional and systematic sampling were used to collect data in 2020 from 229,269 individuals. After exclusion, the valid data from 141,902 adults (86,163 urban and 44,739 rural) were analyzed. We performed t-tests and analyses of variance to ascertain the differences in COVID-19 preventive behaviors according to demographic characteristics, and a post-hoc analysis was conducted using Scheffé’s test. Factors that affected participants’ COVID-19 preventive behaviors were analyzed using multiple regression analyses.

**Results:**

The variables significantly influencing COVID-19 preventive behaviors in urban areas were age, gender, living with two or more people, educational level, monthly household income, working status, influenza vaccination, daily life stress, and perceived threat. In rural areas, age, gender, living with two or more people, education level, influenza vaccination, daily life stress, perceived threat, and perceived social factors were significantly associated with increased COVID-19 preventive behaviors.

**Conclusions:**

Several demographic characteristics were associated with urban and rural residents’ COVID-19-related preventive behaviors. A different approach is needed for the two regions in future policy. Future studies should aim to improve the power of the model and include other factors that may be related to COVID-19 preventive behavior.

## Background

In January 2022, the number of COVID-19 cases in South Korea exceeded 44 million, even though more than 80% of the country had received the first dose of the vaccine [1]. From the early onset of COVID-19, owing to insufficient knowledge and the absence of medication to treat the prevalent symptoms, healthcare professionals recommended preventive actions to reduce the risk of infection [2, 3]. To combat the spread of the virus, keeping a two-meter distance, washing hands, wearing masks publicly, individual quarantine rules, and international lockdowns have become important measures [4]. While they seemed effective at combating the virus, adverse outcomes such as restricting individuals’ physical activities, depression [3], and the negative impact on the social and economic status of households across the country made other safer measures necessary [2].

After 59.5% of the world’s population had received at least one dose of the vaccine [5], many nations tried to return to pre-pandemic life without the noted safety measures. Unfortunately, mutations in the virus meant that the personal quarantine rules had to be reinforced [6]. However, individuals have not only the responsibility to follow public health rules but also the right to take care of their (and their families’) physical and mental well-being by engaging in indispensable social activity [7]. Therefore, it is critical to study preventive health behaviors as they can vary depending on different individual determinants.

**Fig. 1:**
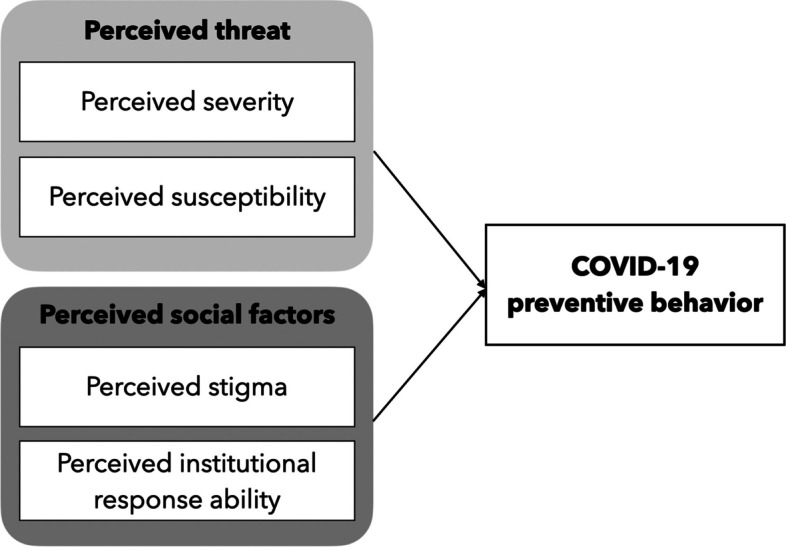
Study framework of the perceived threat and perceived social factors concerning COVID-19 preventive behavior

Although several theories have attempted to explain why individuals do not practice healthy behaviors, the Health Belief Model (HBM) is the critical framework for health preventive behavior. We examined three HBM constructs: perceived threats, perceived severity, and perceived susceptibility [8]. The perceived benefits that affect individual health behavior are caused by an individual’s belief that perceived threats can be reduced, or positive outcomes can result from health behavior; whereas perceived barriers can result from the belief in losses, negative consequences, and costs owing to the behavior [9]. A recent study showed that individuals’ perception of COVID-19 affected their infection preventive behavior [10]. Thus, these selected constructs from the HBM can be used to analyze the demographic, socio-psychological, and structural variables that affect individuals’ health behavior [11]. This study applied this framework to identify the factors influencing COVID-19 preventive behaviors (Fig. 1).

Most people are aware of the COVID-19 threat and behave based on their beliefs [12]. These threats affect the prevention of COVID-19 through the perception people have that they are highly likely to be infected (perceived susceptibility) and that their health can be severely damaged when they fall ill (perceived severity) [9]. Depending on an individual’s perceived sensitivity, the evaluation of other people's health behavior can differ, and the recommended prevention rules are actively followed according to the perceived severity [13, 14]. An awareness of these threats is important because it can predict the intentions behind health behaviors and influence the practice of preventive behaviors [15].

Recently, in previous research on COVID-19 infection preventive behavior, a cross-sectional study conducted in Iran stated that perceived barriers and fatalistic beliefs were more significantly affected than self-efficacy and perceived benefit [16]. In Pakistan, perceived threats significantly affected personal lifestyles and coping with stress in healthcare workers [17]. An international study showed that individuals’ trust in the government concerning their ability to control COVID-19 was significantly associated with infection preventive behaviors adaptation [18]. Another study, which included structural equation modeling, established that perceived severity, perceived susceptibility, subjective norms, perceived health control, and intention—directly and indirectly—affected the preventive behavior of South Korean youths [19]. However, those previous studies have limitations since they only included specific groups of people or focused on individual factors that have not been considered in social and structural contexts for COVID-19 preventive behaviors.

Several countries, including the United Kingdom, the United States, and Brazil, failed to respond adequately to the initial outbreak, underestimating the impact of the virus [20]. From the previous experience of the Severe Acute Respiratory Syndrome (SARS) epidemic, it was acknowledged that trust in government policy and exposure to public health messages can lead to preventive behavior against infectious diseases [21]. Moreover, in research on Middle East Respiratory Syndrome disease in South Korea, health behavior was significantly related to trust in policy and belief in the spread of the disease [22]. In particular, presenting confrontational policy and proper communication, based on scientific evidence, induces related health behaviors and improves trust in the government, institutions, and media [23]. That is, health behavior is affected by the social environment—the higher the trust in government policy, the more people are willing to comply with the system and preventive rules [24]. Thus, it was concluded that it is necessary to understand COVID-19 preventive behavior from this perspective.

Trusting the government is key in a democratic society and can affect the behavior of citizens in a crisis [25]; thus, collectivism may increase individuals’ trust in and compliance to government policies [25]. South Korea has been rated as one of the countries that responded well to the COVID-19 pandemic in the East, where the collectivistic culture is highly critical [26]. Since collectivistic values are known to be prevalent in South Korea, a recent COVID-19 health behavior study stated that perceived stigma can affect individuals’ preventive actions [27]. To verify the research questions, social factors (i.e., the adequacy of the community institutions and neighbors’ response to the pandemic event) were added to the survey.

Using national data collected from all provinces, COVID-19 health behaviors were analyzed by separating urban and rural areas, which reported different results in other countries [16]. Although there are fewer social interactions in rural areas than in urban areas, high infection and mortality rates have been reported owing to low testing and the reluctance to wear masks, and not following government policy [28]. As early reports noted that the concerns and trust in health professionals, which affected preventive behaviors, tended to be lower in rural areas compared to urban ones, it is important to study the level of COVID-19 perception among regions and adherence to prevention rules [29]. Another study stated that in rural areas, the lower the trust in government policy, the lower were residents’ infection preventive behaviors [30]. Therefore, this study was conducted to identify what factors affect individuals’ health behaviors in preventing COVID-19 and whether these differ in distinct regions. From the analysis results, an attempt was made to suggest an effective and practical approach to public health policy.

## Methods

### Study population and data collection

We used data from a community health survey, which has been performed in South Korea annually at public health centers across the country since 2008 to establish and evaluate local health plans, and to produce comparable health statistics of the regions by standardization of the survey system. The community health survey was conducted from August 16 to October 31, 2020, when a trained surveyor directly visited the sample households and conducted face-to-face interviews (computer-assisted personal interviewing) with the participants through a laptop. The survey has 142 items across 18 categories such as smoking, drinking, COVID-19. The target population was adults aged ≥ 19 years as of July 2020. First, based on the number of households by housing type (apartment/detached house) in the district, the sample regions allocated to each district (Dong/Eup/Myeon) were extracted using probability proportional sampling. Second, the sample households were selected using systematic sampling, and an average of five households per sample regions were selected.

We only used data from 2020 after the outbreak of COVID-19. Of the 229 269 cases who participated in the 2020 community health survey, 141,902 cases were used for the final analysis, excluding those who had at least one missing value on the three items with eight questions related to COVID-19 preventive behavior. In South Korea, there are 17 cities and provinces and “Dong, Eup, and Myeon” is an administrative district classified according to city type and the number of residents. For this comparative analysis, 86 163 residents reported living in “Dong”—a regional unit in an urban area—while 55,739 residents reported living in “Eup”/ “Myeon”—a regional unit in a rural area.

### Measures

The variables consisted of four sections: demographic characteristics, perceived threats, perceived social factors, and COVID-19 preventive behaviors. Each item is presented in Table 1.

**Table 1:** Study measures

Variables	Categories	Reference
**Demographic characteristics**		
	· Age (reclassified as 19–29, 30–49, 50–69, ≥ 70 years) · Gender (male, female) · Education (under elementary school, middle/high school, associate/bachelor’s degree, master’s or higher degree) · Monthly household income (reclassified as less than 100, 101–300, 301–500, ≥ 501; ten thousand won unit) · Type of household (single-person household, household with two or more members) · Working status (yes/no) · Influenza vaccination (yes/no) · Daily life stress (seldom felt, felt a little, felt much, extremely felt)	Li et al. [31] Beaudoin et al. [32] Gunderson et al. [33] Tang et al. [34] Peterson et al. [35]
**COVID-19 preventive behaviors**		
Wearing a mask	· Did you cover your mouth and nose with your sleeve when sneezing or coughing? · Did you wear a mask in indoor facilities used by unspecified people? · Did you wear a mask if it is difficult to keep a distance of more than two meters between people outdoors?	Li et al. [31] Graupensperger et al [36] Monnig et al. [37]
Social distancing	· Did you maintain a two-meter distance between people for your health? · Did you refrain from going outside/meetings/events?	
Hand washing	· How often did you wash your hands after returning from outside? · Did you thoroughly wash your hands under running water for at least 30 s? · How often do you use soap or hand sanitizer when washing your hands?	
**Perceived threat**		
Perceived severity	· I am concerned that I will die if I become infected with COVID-19 · I am concerned that the COVID-19 epidemic will cause economic damage to my family and myself including losing my job or difficulty in finding a job	Rayani et al. [38] Beaudoin et al. [32] Fujii et al. [39]
Perceived susceptibility	· I am concerned that I will be infected with COVID-19 · I am concerned that my family and other vulnerable people (older people, infants, and patients) will become infected with COVID-19	
**Perceived social factors**		
Perceived stigma	· I am concerned that I will be criticized or harmed by those around me if I am infected with COVID-19	Hong et al. [27]
Perceived institutional response ability	· Do you think the COVID-19 response ability of each of the following institutions is appropriate? - government including the Ministry of Health and Welfare and the Korea Centers for Disease Control and Prevention - mass media such as broadcasting and newspapers - local medical institutions - neighbors and co-workers	Chen et al. [25] Rieger et al. [24] Liu & Mesch [40]

#### Demographic characteristics

Variables affecting COVID-19 preventive behaviors were selected as demographic characteristics. There were eight items: age (reclassified as 19–29, 30–49, 50–69, ≥ 70 years), gender (male, female), education (under elementary school, middle/high school, associate/bachelor’s degree, master’s or higher degree), monthly household income (reclassified as less than 100, 101–300, 301–500, and 501 or more; ten thousand won unit), type of household (single-person household, household with two or more members), influenza vaccination (yes/no), working status (yes/no), and daily life stress. Daily life stress was measured with a single question: “How much stress do you usually feel in your daily life?” Responses were made with a four-point Likert scale ranging from 1 “seldomly felt” to 4 for “extremely felt,” with higher scores indicating higher levels of stress.

#### COVID-19 preventive behaviors

COVID-19 preventive behaviors were based on the guidelines of World Health Organization, Centers for Disease Control and Prevention, and prior literature [31, 36, 37]. Three items are assessed with eight questions: hand washing, wearing a mask, and social distancing. Regarding wearing a mask, three questions were selected (e.g., “Did you wear a mask when it was not possible to keep the distance of more than 2 m between people outdoors?”). Responses were 1: *strongly agree*, 2: *agree*, or 3: *disagree*. Social distancing consisted of two questions (e.g., “Did you practice the following social distancing procedures during the past week; that is, maintaining a two-meter distance between people outdoors and refraining from going outside/meetings/events?”). Responses were 1: *strongly agree*, 2: *agree*, or 3: *disagree*. The average scores coded in reverse were used for wearing a mask and social distancing. Hand washing included three questions (e.g., “Did you thoroughly wash your hands under running water for at least 30 s when doing regular washing?”). Responses were 1: *always*, 2: *often*, 3: *sometimes*, or 4: *almost never* (reclassified as: 3 (*always*), 2 (*often*), and 1 (*sometimes/almost never*)). For the eight questions, the average scores were used, which represented higher scores and greater engagement in COVID-19 preventive behaviors. The range of COVID-19 preventive behaviors’ scores was from 1 to 3.

#### Perceived threat

Perceived threat consists of perceived severity and perceived susceptibility. Perceived severity was based on two statements: “I am concerned that I will die if I become infected with COVID-19” and “I am concerned that the COVID-19 epidemic will cause economic damage to my family and myself including losing my job or difficulty in finding a job” (1: *strongly agree*, 2: *agree*, 3: *neutral*, 4: *disagree*, or 5: *never*). Perceived severity scores ranged from 1 to 5.

Perceived susceptibility was assessed through two statements: “I am concerned that I will be infected with COVID-19” and “I am concerned that my family and other vulnerable people (older people, infants, and patients) will become infected with COVID-19” (1: *strongly agree*, 2: *agree*, 3: *neutral*, 4: *disagree*, or 5: *never*). The average scores coded in reverse were used; the higher the scores, the greater the severity and susceptibility. Perceived susceptibility scores ranged from 1 to 5.

#### Perceived social factors

Perceived social factors consisted of perceived stigma and perceived institutional response ability. Perceived stigma was assessed through the sentence, “I am concerned that I will be criticized or harmed by those around me if I am infected with COVID-19” (1: *strongly agree*, 2: *agree*, 3: *neutral*, 4: *disagree*, or 5: *never*). Perceived institutional response ability was assessed through the question, “Do you think the COVID-19 response ability of each of the following institutions (government including the Ministry of Health and Welfare and the Korea Centers for Disease Control and Prevention/mass media such as broadcasting and newspapers/local medical institutions/neighbors and co-workers) is appropriate?” (1: *strongly agree*, 2: *agree*, 3: *neutral*, 4: *disagree*, or 5: *never*). All perceived social factors were coded in reverse and analyzed using average scores. Perceived social factors scores ranged from 1 to 5. The higher the score, the higher the perceived stigma of trust in institutional response ability.

### Data analysis

We analyzed the data using IBM SPSS Statistics 25.0 (Armonk, NY, USA), with significance set at 0.05. A descriptive analysis was conducted to examine demographic characteristics, perceived threat, perceived social factors, and the degree of COVID-19 preventive behaviors. We performed t-tests and analyses of variance to ascertain the differences in COVID-19 preventive behaviors according to the demographic characteristics, and a post-hoc analysis using Scheffé’s test. Factors that affect participants’ COVID-19 preventive behaviors were analyzed using multiple regression analyses. Cronbach’s αs were calculated to verify the reliability of the items.

## Ethical considerations

The community health survey was conducted after approval by the Medical Research Ethics Review Committee of the Korea Centers for Disease Control and Prevention (KCDC). It was conducted after obtaining informed consent from all survey participants prior to data collection. The raw data used in this study were provided as non-identifiable information after obtaining approval from the KCDC through the community health survey website (https://chs.kdca.go.kr/chs/index.do). In addition, this study was exempted from obtaining consent from the appropriate institutional review board (no. Ewha-202203–0028-01).

## Results

### Differences between urban and rural COVID-19 preventive behaviors according to demographic characteristics

Table 2 shows the differences between urban and rural COVID-19 preventive behaviors according to demographic characteristics. In both urban and rural areas, women’s COVID-19 preventive behaviors were higher than those of men (*p* < 0.001).

**Table 2:** Difference in COVID-19 prevention behavior according to demographics in urban and rural areas

Variables	Urban (*N* = 86,163)				Rural (*N* = 55,739)			
	**N (%)**	**M (SD)**	**F/t**	***p* (Scheffé test)**	**N (%)**	**M (SD)**	**F/t**	***p* (Scheffé test)**
**Gender**								
Male	39,331 (45.6)	2.62 (0.32)	-44.366	< .001	25,654 (46.0)	2.54 (0.37)	-21.651	< .001
Female	46,832 (54.4)	2.71 (0.29)			30,085 (54.0)	2.61 (0.36)		
**Age**								
19-29^a^	14,288 (16.6)	2.65 (0.30)	191.280	< .001 (d < a < c < b)	4586 (8.2)	2.64 (0.33)	726.399	< .001 (a,b > c > d)
30-49^b^	29,366 (34.1)	2.69 (0.29)			12,489 (22.4)	2.65 (0.35)		
50-69^c^	30,832 (35.8)	2.67 (0.31)			23,294 (41.8)	2.60 (0.35)		
≥ 70^d^	11,677 (13.6)	2.62 (0.34)			15,370 (27.6)	2.46 (0.40)		
Education^*^								
Under elementary school^a^	9166 (10.6)	2.60 (0.34)	244.077	< .001 (a < b < c < d)	16,297 (29.2)	2.47 (0.40)	829,788	< .001 (a < b < c < d)
Middle/high school^b^	32,884 (38.2)	2.66 (0.32)			23,516 (42.2)	2.59 (0.36)		
Associate/bachelor’s degree^c^	39,653 (46.0)	2.69 (0.29)			14,544 (26.1)	2.66 (0.32)		
Master and higher^d^	4310 (5.0)	2.72 (0.27)			1274 (2.3)	2.71 (0.29)		
Income (monthly)^*^								
≤ 100^a^	6106 (7.1)	2.63 (0.33)	85.728	< .001 (a < b < c < d)	9387 (16.8)	2.48 (0.39)	270.289	< .011 (c,d > b > a)
101-300^b^	20,371 (23.6)	2.65 (0.32)			14,713 (26.4)	2.56 (0.37)		
301-500^c^	20,031 (23.2)	2.67 (0.31)			9768 (17.5)	2.61 (0.35)		
≥ 500^d^	25,356 (29.4)	2.69 (0.30)			8211 (14.7)	2.62 (0.34)		
Household type^*^								
Single person households	10,965 (12.7)	2.62 (0.33)	-14.941	< .001	9202 (16.5)	2.51 (0.39)	-18.286	< .001
≥ 2 person households	75,187 (87.3)	2.67 (0.30)			46,537 (83.5)	2.59 (0.36)		
Working status^*^								
Working	52,291 (60.7)	2.67 (0.30)	-1.378	0.168	36,273 (65.1)	2.56 (0.38)	-8.742	< .001
Not working	33,853 (39.3)	2.67 (0.31)			19,440 (34.9)	2.59 (0.36)		
Influenza vaccination^*^								
Vaccinated	43,776 (50.8)	2.68 (0.31)	-14.414	< .001	35,161 (63.1)	2.60 (0.35)	11.899	< .001
Not vaccinated	42,238 (49.0)	2.65 (0.31)			20,493 (36.8)	2.56 (0.38)		
Stress^*^								
Seldomly felt stressed^a^	16,291 (18.9)	2.67 (0.31)	4.158	0.006 (a,b,c < d)	16,575 (29.7)	2.55 (0.38)	47.247	< .001 (a < b < c < d)
Felt a little stressed^b^	48,026 (55.7)	2.67 (0.31)			28,724 (51.5)	2.58 (0.36)		
Felt much stressed^c^	18,960 (22.0)	2.67 (0.30)			9120 (16.4)	2.60 (0.35)		
Extremely felt stressed^d^	2880 (3.3)	2.69 (0.30)			1308 (2.3)	2.62 (0.36)		

^*^All missing values are included

^a,b,c^ and ^d^ represented the variable results of the scheffe test in order

Concerning age, in urban areas, COVID-19 preventive behaviors were higher in the order of 30–49, 50–69, 19–29, and ≥ 70 years (*p* < 0.001). In rural areas, there was no difference between those aged 19–49 years; however, they had higher preventive behaviors than those aged ≥ 50 years (*p* < 0.001). In both areas, the higher the education level and the higher the monthly household income, the higher the preventive behaviors (*p* < 0.001); however, there was no significant difference between the 301–500 and ≥ 500 income groups in rural areas. In both areas, preventive behaviors were higher in households with two or more people (*p* < 0.001) as compared to their counterparts. There was no difference between the groups in terms of working status in urban areas; however, in rural areas, preventive behaviors in those who did not (vs. did) engage in economic activity were higher (*p* < 0.001). In both areas, the vaccinated group showed higher preventive behaviors than did the unvaccinated (*p* < 0.001). In urban areas, the group experiencing a high amount of stress showed higher preventive behaviors than the other three groups (*p* < 0.001). In rural areas, the higher the stress, the higher the preventive behaviors (*p* < 0.001).

Frequency distribution of COVID-19 preventive behaviors in urban and rural areas.

Table 3 shows the frequency distribution of COVID-19 preventive behaviors in urban and rural areas. Among the COVID-19 preventive behaviors in both areas, the most frequently answered “always” was for the question, “Did you wear a mask in indoor facilities used by unspecified people?” (86.0 vs. 79.4%), which was followed by two questions: “Did you wear a mask when it was not possible to keep the distance of more than 2 m between people outdoors?” (85.1 vs. 75.9%), and “How often do you wash your hands after returning home?” (82.9 vs. 70.3%). However, for all the three questions, the ratio was higher in urban areas than in rural areas.

**Table 3:** Frequency distribution of COVID-19 preventive behaviors in Urban and rural area

	**Urban (*n* = 86 163)**			**Rural (*n* = 55 739)**		
**Variables**	**Not agree/never n (%)**	**Agree/sometimes n (%)**	**Strongly agree/always n (%)**	**Not agree/never n (%)**	**Agree/sometimes n (%)**	**Strongly agree/always n (%)**
Did you cover your mouth and nose with your sleeve when sneezing or coughing?	3 780 (4.4)	26 373 (30.6)	56 010 (65.0)	4 170 (7.5)	20 435 (36.7)	31 134 (55.9)
Did you wear a mask in indoor facilities used by unspecified people?	282 (0.3)	11 744 (13.6)	74 137 (86.0)	313 (0.6)	11 167 (20.0)	44 259 (79.4)
Did you wear a mask if it is difficult to keep a distance of more than two meters between people outdoors?	443 (0.5)	12 398 (14.4)	73 322 (85.1)	996 (1.8)	12 451 (22.3)	42 292 (75.9)
Did you maintain a two-meter distance between people for your health?	3 542 (4.1)	28 864 (33.5)	53 757 (62.4)	2 118 (3.8)	20 031 (35.9)	33 590 (60.3)
Did you refrain from going outside/meetings/events?	2 285 (2.7)	29 247 (33.9)	54 631 (63.4)	1 192 (2.1)	19 509 (35.0)	35 038 (62.9)
How often did you wash your hands after returning from outside?	1 621 (1.9)	13 122 (15.2)	71 420 (82.9)	2 777 (5.0)	13 774 (24.7)	39 188 (70.3)
Did you thoroughly wash your hands under running water for at least 30 s?	12 093 (14.0)	29 063 (33.7)	45 007 (52.2)	9 806 (17.6)	19 423 (34.8)	26 510 (47.6)

### Frequency distribution of model constructs in urban and rural areas

Table 4 presents the frequency distribution and reliability of the urban and rural model constructs. Perceived threat (3.85 vs. 4.00) and perceived social factors (3.88 vs. 4.05) were higher in rural in comparison to urban areas; however, COVID-19 preventive behaviors were higher in urban than in rural areas (2.67 vs. 2.58). Cronbach’s αs for perceived threat were 0.738 for urban and 0.776 for rural, Cronbach’s αs for perceived social factor were 0.678 for urban and 0.751 for rural, and Cronbach’s αs for COVID-19 preventive behaviors were 0.715 for urban and 0.773 for rural.

**Table 4:** Frequency distribution of model constructs in urban and rural areas

	**Urban (*n* = 86 163)**		**Rural (*n* = 55 739)**	
**Variables (n)**	Mean (SD)	Cronbach’s α	Mean (SD)	Cronbach’s α
**Perceived threat (4)**	3.85 (0.76)	0.738	4.00 (0.78)	0.776
Perceived severity (2)	3.62 (0.89)	0.498	3.84 (0.90)	0.543
Perceived susceptibility (2)	4.08 (0.80)	0.635	4.17 (0.80)	0.652
**Perceived social factor (5)**	3.88 (0.62)	0.678	4.05 (0.64)	0.751
Perceived social norms (1)	3.94 (1.00)	-	4.12 (0.96)	-
Perceived response capacity in institutions (4)	3.81 (0.64)	0.781	3.98 (0.67)	0.834
**COVID-19 preventive behavior (8)**	2.67 (0.31)	0.715	2.58 (0.37)	0.773
Wearing a mask (3)	2.77 (0.35)	0.677	2.67 (0.41)	0.709
Social distance (2)	2.45 (0.61)	0.621	2.46 (0.41)	0.671
Handwashing (3)	2.61 (0.44)	0.580	2.47 (0.53)	0.677

*COVID-19* Coronavirus 2019, *SD* Standard deviation

### Predictors affecting COVID-19 preventive behaviors in urban and rural areas

Table 5 shows predictors influencing urban and rural COVID-19 preventive behaviors. Consequent to the multiple regression analysis, the model explained 7.0% of the variance of COVID-19 preventive behaviors in urban areas. The variables influencing COVID-19 preventive behaviors were age (β = -0.018, *p* < 0.001), gender (female) (β = 0.144, *p* < 0.001), household type having two or more members (β = 0.033, *p* < 0.001), education level (β = 0.136, *p* < 0.001), monthly household income (β = 0.001, *p* = 0.012), working status (β = 0.022, *p* < . 001), influenza vaccination (β = 0.050, *p* < 0.001), daily life stress (β = -0.025, *p* < 0.001), perceived threat (β = 0.022, *p* < 0.001), and perceived social factors (β = 0.089, *p* < 0.001); all of these had significant effects on COVID-19 preventive behaviors (F = 543.392, *p* < 0.001).

**Table 5:** Effect in COVID-19 preventive behaviors according to demographics, model constructs in urban area

	**Urban (*N* = 86,163)**					**Rural (*N* = 55,739)**				
**Variables**	**B**	**SE**	**β**	**t**	**p**	**B**	**SE**	**β**	**t**	**p**
(constant)	2.104	0.012		182.892	< .001	1.916	0.019		101.075	< .001
Age	0.000	0.000	-0.018	-3.763	< .001	-0.003	0.000	-0.124	-17.663	< .001
Gender^a^	0.090	0.002	0.144	38.336	< .001	0.081	0.004	0.110	22.621	< .001
Household type^b^	0.030	0.004	0.033	8.290	< .001	0.029	0.005	0.030	5.956	< .001
Education	0.056	0.002	0.136	29.646	< .001	0.090	0.003	0.196	29.997	< .001
Income(monthly)	0.004	0.001	0.011	2.522	0.012	-0.001	0.002	-0.002	-0.312	0.755
Working status^c^	0.014	0.002	0.022	5.633	< .001	0.005	0.004	0.007	1.465	0.143
Influenza Vaccination^d^	0.031	0.002	0.050	12.682	< .001	0.020	0.004	0.026	4.991	< .001
Stress	-0.011	0.002	-0.025	-6.665	< .001	-0.012	0.002	-0.024	-4.986	< .001
Perceived threat	0.048	0.002	0.117	27.082	< .001	0.052	0.003	0.109	18.890	< .001
Perceived social factors	0.044	0.002	0.089	20.996	< .001	0.089	0.003	0.151	26.346	< .001
	adj. R^2^ = .070, *F* = 543.392, *p* < *.*001					adj. R^2^ = .116, *F* = 550.425, *p* < *.*001				

^a^Dummy variable(0 = male)

^b^Dummy variable (0 = one person household)

^c^Dummy variable (0 = Not working)

^d^Dummy variable (0 = Not vaccinated)

In rural areas, the model explained 11.6% of the variance in COVID-19 preventive behaviors. The results showed that age (β = -0.124, *p* < 0.001), gender (female) (β = 0.110, *p* < 0.001), household type with two or more members (β = 0.030, *p* < 0.001), education level (β = 0.196, *p* < 0.001), influenza vaccination (β = 0.026, *p* < 0.001), daily life stress (β = -0.024, *p* < 0.001), perceived threat (β = 0.109, *p* < 0.001), and perceived social factors (β = 0.151, *p* < 0.001) were associated with higher COVID-19 preventive behaviors, and these variables had significant effects on COVID-19 preventive behaviors (F = 550.425, *p* < 0.001). The associations with monthly household income and employment status were non-significant.

## Discussion

This study identified factors influencing COVID-19 preventive behaviors according to participants’ urban and rural residence by using data from the 2020 community health survey in South Korea. This study cited the constructs of HBM to explain individual health preventive behavior and further applied perceived threats and perceived social factors.

Our findings revealed that, in both urban and rural areas, women rather than men had higher preventive behavior, and the higher their educational background and economic power, the greater the COVID-19 infection preventive behavior. Studies have shown that women generally have a higher understanding and knowledge of diseases than do men [41], and they play a more prominent role in taking care of their families in dangerous situations such as natural disasters [42]. In addition, women are more concerned and afraid of COVID-19 than men, which explains why they actively engage in infection preventive behaviors [43]. Men were found to be three times more likely to be admitted to the intensive care unit than women [44], and studies have confirmed that COVID-19 infections have serious consequences for men [45].

Income and education levels are closely related to infection preventive behavior, and the difference in preventive behavior according to income can be expected in that people with high education levels have jobs with high incomes, and those with higher incomes can obtain better accurate health information than their counterparts [46]. During the 2002 SARS epidemic, high-income was associated with accurate information access and preventive actions related to infectious diseases [47]. This study found that COVID-19 preventive behavior was performed more when family members were living together rather than in a single-person household. This tendency seems to be because single-person families do not have to worry about children or older adults who are highly vulnerable to the disease [48]. Even in the 2009 H1N1 influenza outbreak, people with cohabitation families participated highly actively in preventive actions to avoid infection [49].

In this study, the 30–49 years age range group performed preventive behavior better in urban areas, while the 19–29 and 30–49 years age range groups performed preventive behavior better in rural areas. There were no significant differences in those aged 70 years or older in both areas, most of whom did not adopt adequate preventive measures. This result is consistent with that of previous studies that demonstrated that older adults perceive a higher risk of COVID-19 but perform fewer preventive actions because they are less concerned about the severity of COVID-19 [50]. However, since the older adults have a higher risk of COVID-19 infection, prior studies have also confirmed higher preventive behavior than middle-aged or young people [51]. Further studies should analyze factors according to age groups and mediate appropriate infection preventive behavior. In contrast to the results of this study, previous research has determined that young people are less likely to be infected with COVID-19 or develop serious symptoms or complications than their older counterparts [52]. Therefore, they do not observe preventive behavior and ignore the importance of the guidelines [53]. In this study, it is assumed that the 30–49 years age range group actively participated in preventive actions owing to economic concerns because this age group is typically responsible for economic activities.

In this study, both urban and rural participants felt “very much” stressed about COVID-19. Research has established that anxiety about the severity and transmission of the disease changed habits, induced concerns about job security and subsequent financial problems, and frequent fluctuations in the isolation period and social distancing caused stress [12]. Life-threatening public health emergencies can lead to depression and anxiety disorders, particularly in vulnerable groups with reduced immune function [54]. Therefore, proper management of people experiencing physical and mental health concerns at all stages of infectious disease management is needed [55]. Healthcare in rural areas with high proportions of vulnerable populations—such as older adults—but low medical infrastructure should not be ignored.

In the case of flu vaccines, people who were vaccinated against the flu in both urban and rural areas showed COVID-19 preventive behavior. Although it is difficult to accurately compare urban and rural flu vaccinations with COVID-19 vaccination owing to the lack of adequate research, follow-up studies on the link between COVID-19 vaccination and flu vaccination are needed [56]. The high participation in COVID-19 preventive behavior according to gender, income level, presence or absence of cohabitation families, and education level corroborated the findings of previous studies, but preventive behavior by age showed different results.

In this study, most people practiced COVID-19 infection preventive behavior, and more than 50% expressed “very much” concerning all areas of infection prevention rules in urban and rural areas. Studies conducted in Iran and Hong Kong also confirmed that the compliance rate for infection preventive behavior was high, thereby supporting the results of this study [16, 57]. Looking at the perceived threats according to the theoretical framework applied in this study, both urban and rural areas were concerned that vulnerable people (older adults, infants, patients) would be infected with COVID-19, and they were concerned about the economic ramifications. In particular, in rural areas, older adults account for a large proportion of the residents; therefore, there is a high possibility that they will not receive immediate treatment even if they are infected with COVID-19 owing to a lack of suitable medical facilities [48].

Looking at the perceived stigma among perceived social factors, studies generally show that people tend to shift the responsibility to those who have not implemented preventive measures in the event of a new infectious disease epidemic [58]. The Shincheonji Church of Jesus, one of South Korean’s religious sects, was responsible for a church-wide infection in February 2020, which is a representative example of stigma and perception of a specific group. In previous studies, 77.4% stated, “I am reluctant to belong to a specific religion, Shincheonji” [59]. In a study by Jang and Sohn [60], the reason for wearing a mask was that it is perceivable by others; those who did not wear it would be criticized by others. Like previous research, this study also confirmed that perceived stigma is an important factor that causes certain actions to be performed; however, additional research is needed as higher negative stigma can increase anxiety and social tension.

Concerning trust, most participants responded positively. Studies have shown that factors that increase risk awareness and preventive behavior related to COVID-19 include not only direct experience but also trust and pro-social values in government and community healthcare [61]. In addition, existing literature noted that perception drives behavioral changes and emphasized that media plays an important role in providing and educating the public with information related to new infectious diseases such as COVID-19 [32]. Recently, owing to the high consumption of health information through social networking services, appropriate infection prevention education is required through said services, and individuals’ ability to evaluate accurate information is vital [62].

Perceived threats and perceived social factors were high in rural areas, but COVID-19 preventive behavior was lower in rural areas than in urban areas. Rural areas have fewer medical facilities than cities, making it difficult to respond immediately to COVID-19 [48], and rural workers often lose their jobs when infected with COVID-19 because, unlike cities, there are many jobs that are difficult to perform at home. In addition, according to previous studies, we confirmed that living with others has a positive effect on the infection preventive behavior of the elderly in rural areas [63], which is considered to be sensitive to the support and evaluation of close acquaintances. However, the COVID-19 preventive behaviors were relatively low in rural areas owing to their lower level of information evaluation ability and positive attitude toward the efficiency of performing preventive actions [46]. A study found that rural areas have low health preventive behavior because of the high concentration of conservative voters who are reluctant to wear masks [48]. Taken together, to increase COVID-19 preventive behavior in rural areas, various factors that affect such behavior in rural areas should be identified through follow-up studies, and infection prevention education and policies should be implemented accordingly.

Finally, to identify the factors related to the model used in this study of COVID-19 preventive behavior, the effect of each factor on health behavior through multiple regression analysis was 7% in cities and 11.6% in rural areas. In the case of cities, gender was the largest related factor. In the case of rural areas, education level was the most related factor. The results confirmed that each variable had a significant effect on infection preventive behavior; however, the R^2^ values were low. Although the power of the model is low owing to limited survey data, other factors can be added in further research.

Several questions were selected and analyzed to compare COVID-19 preventive behaviors in urban and rural areas; therefore, there is a limit to generalizing the results. In addition, since this study was conducted using community health survey data during the COVID-19 pandemic, repeated studies are needed to determine whether the model can be applied to other infectious disease studies. Moreover, research on infection preventive behavior by age is necessary, and it seems that policy establishment, public promotion, and education are critical.

## Conclusions

This study is one of the few to apply national data and theoretical models to COVID-19 preventive behavior in South Korea, including both urban and rural areas. We began with an assumption that each characteristic of urban and rural areas will affect COVID-19 preventive behavior while simultaneously identifying why individuals do not behave properly in relation to their health. The results showed that several demographic characteristics of urban and rural residents were associated with COVID-19 preventive behaviors; thus, there should be a distinct approach that considers region in policy formulation. In particular, the study is significant as not many other measures were suggested besides compliance with personal quarantine rules during the early phase of the pandemic in 2020. The used model identified social factors, including perceived stigma and perceived institutional response ability, as vital. We hope that these factors will be considered in further analysis of infection preventive behavior and in future health crises.

## Data Availability

The data which support the findings of this study is available from the Korean Disease Control and Prevention Agency; however, permission is required to access the data under license only for research purpose—it not publicly available (https://chs.kdca.go.kr). The raw data supporting the conclusion of this study will be available by the corresponding author, without undue reservation.

## Abbreviations

COVID-19: Coronavirus disease 2019
HBM: Health Belief Model
KCDC: Korea Centers for Disease Control and Prevention

## References

[1] Coronavirus(COVID-19), Republic of Korea. Korean disease control and prevention agency; 2022. http://ncov.mohw.go.kr/bdBoardList_Real.do.Accessed. Accessed 20 Jan 2022.

[2] Gostin LO, Wiley LF (2020). Governmental public health powers during the COVID-19 pandemic: stay-at-home orders, business closures, and travel restrictions. JAMA.

[3] Knell G, Robertson MC, Dooley EE, Burford K, Mendez KS (2020). Health behavior changes during COVID-19 pandemic and subsequent “stay-at-home” orders. Int J Environ Res Public Health.

[4] Wong LP, Alias H, Wong PF, Lee HY, Abubakar S (2020). The use of the health belief model to assess predictors of intent to receive the COVID-19 vaccine and willingness to pay. Hum Vaccin Immunother.

[5] Our world data. Global change data. LaB. 2021. https://ourworldindata.org/. Accessed 20 Jan 2022.

[6] Van den Broucke S (2020). Why health promotion matters to the COVID-19 pandemic, and vice versa. Health Promot Int Oxford University Press.

[7] Denford S, Morton KS, Lambert H, Zhang J, Smith LE, Rubin GJ (2021). Understanding patterns of adherence to COVID-19 mitigation measures: a qualitative interview study. J Public Health (Oxf).

[8] Rosenstock IM. Health belief model. Encyclopedia of psychology. In: Kazdin, Alan E editors. Washington, DC, US: American Psychological Association; New York, NY, US: Oxford University Press; 2002:78–80.

[9] Kim J, Cho J (2019). Investigation of effects of individuals’ social viewing of fine dust information obtained through social media on behavioral intentions of disease prevention: application of health beliefs model. Korea J Broadcast Telecommunication Stud.

[10] Van Lissa CJ, Stroebe W, vanDellen MR, Leander NP, Agostini M, Draws T (2022). Using machine learning to identify important predictors of COVID-19 infection prevention behaviors during the early phase of the pandemic. Patterns (N Y).

[11] Champion VL, Skinner CS. The health belief model. In: Health behavior and health education: theory, research, and practice, 4. Glanz, K, Rimer, BK, Viswanath, K, editors; 2008. p. 45–65.

[12] Park CL, Ressell BS, Fendrich M, Finkelstein-Fox L, Hutchison M, Becker J (2020). Americans’ COVID-19 stress, coping, and adherence to CDC guidelines. J Gen Intern Med.

[13] Luquis RR, Kensinger WS (2019). Applying the health belief model to assess prevention services among young adults. Int J Health Promot Educ.

[14] Sim SW, Moey KSP, Tan NC (2014). The use of facemasks to prevent respiratory infection: a literature review in the context of the Health Belief Model. Singapore Med J.

[15] Lau BH-P, Chan CL-W, Ng SM (2020). Self-compassion buffers the adverse mental health impacts of COVID-19-related threats: results from a cross-sectional survey at the first peak of Hong Kong's outbreak. Front Psychiatry.

[16] Shahnazi H, Ahmadi-Livani M, Pahlavanzadeh B, Rajabi A, Hamrah MS, Charkazi A (2020). Assessing preventive health behaviors from COVID-19: a cross sectional study with health belief model in Golestan Province. Northern Iran Infect Dis Poverty.

[17] Mukhtar S (2020). Mental health and emotional impact of COVID-19: applying health belief model for medical staff to general public of Pakistan. Brain Behavior Immuniy.

[18] Han Q, Zheng B, Cristea M, Agostini M, Bélanger JJ, Gützkow B (2021). Trust in government regarding COVID-19 and its associations with preventive health behaviour and prosocial behaviour during the pandemic: a cross-sectional and longitudinal study. Psychol Med.

[19] Park S, Oh S (2022). Factors associated with preventive behaviors for COVID-19 among adolescents in South Korea. J Pediatr Nurs.

[20] Vacondio M, Priolo G, Dickert S, Bonini N (2021). Worry, perceived threat and media communication as predictors of self-protective behaviors during the COVID-19 outbreak in Europe. Front Psychol.

[21] Plough A, Bristow B, Fielding J, Caldwell S, Khan S (2011). Pandemics and health equity: lessons learned from the H1N1 response in los Angeles County. J Public Health Manag Pract.

[22] Hong D, Jun J (2020). Effects of government trust on prevention intention of college students. J Pract Res Advertising Public Relat.

[23] Kreps SE, Kriner DL (2020). Model uncertainty, political contestation, and public trust in science: evidence from the COVID-19 pandemic. Sci Adv.

[24] Rieger MO, Wang M (2022). Trust in government actions during the COVID-19 crisis. Soc Indic Res.

[25] Chen D, Peng D, Rieger MO, Wang M (2021). Institutional and cultural determinants of speed of government responses during COVID-19 pandemic. Humanit Soc Sci Commun.

[26] Kasdan DO, Campbell JW (2020). Dataveillant collectivism and the coronavirus in Korea: values, biases, and socio-cultural foundations of containment efforts. Admin Theor Prax.

[27] Hong D-Y, Jeon M-A, Cho C-H (2021). Predicting preventive behavior intention in COVID-19 pandemic context: application of social variables to health belief model. J Korea Contents Assoc.

[28] Callaghan T, Lueck JA, Trujillo KL, Ferdinand AO (2021). Rural and urban differences in COVID-19 prevention behaviors. J Rural Health.

[29] Ranscombe P (2020). Rural areas at risk during COVID-19 pandemic. Lancet Infect Dis.

[30] Ridenhour BJ, Sarathchandra D, Seamon E, Brown H, Leung FY, Johnson-Leon M (2021). Effects of trust, risk perception, and health behavior on COVID-19 disease burden: evidence from a multi-state US survey.

[31] Li S, Feng B, Liao W, Pan W (2020). Internet use, risk awareness, and demographic characteristics associated with engagement in preventive behaviors and testing: cross-sectional survey on COVID-19 in the United States. J Med Internet Res.

[32] Beaudoin CE, Hong T (2021). Predictors of COVID-19 preventive perceptions and behaviors among millennials: two cross-sectional survey studies. J Med Internet Res.

[33] Gunderson J, Mitchell D, Reid K, Jordan M (2021). COVID-19 information-seeking and prevention behaviors in Florida, April 2020. Prev Chronic Dis.

[34] Tang CC, Chen H, Wu WW (2021). Factors influencing the protective behavior of individuals during COVID-19: a transnational survey. Sci Rep.

[35] Peterson LM, Helweg-Larsen M, Dimuccio S (2021). Descriptive norms and prototypes predict COVID-19 prevention cognitions and behaviors in the United States: applying the prototype willingness model to pandemic mitigation. Ann Behav Med.

[36] Graupensperger S, Lee CM, Larimer ME (2021). Young adults underestimate how well peers adhere to COVID-19 preventive behavioral guidelines. J Prim Prev.

[37] Monnig MA, Treloar Padovano H, Sokolovsky AW, Decost G, Aston ER, Haass-Koffler CL (2021). Association of substance use with behavioral adherence to centers for disease control and prevention guidelines for COVID-19 mitigation: cross-sectional web-based survey. JMIR Public Health Surveill.

[38] Rayani M, Rayani S, Najafi-Sharjabad F (2021). COVID-19-related knowledge, risk perception, information seeking, and adherence to preventive behaviors among undergraduate students, southern Iran. Environ Sci Pollut Res Int.

[39] Fujii R, Suzuki K, Niimi J (2021). Public perceptions, individual characteristics, and preventive behaviors for COVID-19 in six countries: a cross-sectional study. Environ Health Prev Med.

[40] Liu XJ, Mesch GS (2020). The adoption of preventive behaviors during the COVID-19 pandemic in China and Israel. Int J Environ Res Public Health.

[41] Mouchtouri VA, Papagiannis D, Katsioulis A, Rachiotis G, Dafopoulos K, Hadjichristodoulou C (2017). Knowledge, attitudes, and practices about the prevention of mosquito bites and Zika virus disease in pregnant women in Greece. Int J Environ Res Public Health.

[42] Tyler M, Fairbrother P (2018). Gender, households, and decision-making for wildfire safety. Disasters.

[43] Bronfman N, Repetto P, Cordón P, Castañeda J, Cisternas P (2021). Gender differences on psychosocial factors affecting COVID-19 preventive behaviors. Sustainability.

[44] Peckham H, de Gruijter NM, Raine C, Radziszewska A, Ciurtin C, Wedderburn LR (2020). Male sex identified by global COVID-19 meta-analysis as a risk factor for death and ITU admission. Nat Commun.

[45] Zheng Z, Peng F, Xu B, Zhao J, Liu H, Peng J (2020). Risk factors of critical & mortal COVID-19 cases: a systematic literature review and meta-analysis. J Infect.

[46] Chen X, Chen H (2020). Differences in preventive behaviors of COVID-19 between urban and rural residents: lessons learned from a cross-sectional study in China. Int J Environ Res Public Health.

[47] Des Jarlais DC, Galea S, Tracy M, Tross S, Vlahov D (2006). Stigmatization of newly emerging infectious diseases: AIDS and SARS. Am J Public Health.

[48] Peters DJ (2020). Community susceptibility and resiliency to COVID-19 across the rural-urban continuum in the United States. J Rural Health.

[49] Ibuka Y, Chapman GB, Meyers LA, Li M, Galvani AP (2010). The dynamics of risk perceptions and precautionary behavior in response to 2009 (H1N1) pandemic influenza. BMC Infect Dis.

[50] Barber SJ, Kim H (2021). COVID-19 worries and behavior changes in older and younger men and women. J Gerontol B Psychol Sci Soc Sci.

[51] Haischer MH, Beilfuss R, Hart MR, Opielinski L, Wrucke D, Zirgaitis G (2020). Who is wearing a mask? gender-, age-, and location-related differences during the COVID-19 pandemic. PLoS One.

[52] Atchison CJ, Bowman L, Vrinten C, Redd R, Pristera P, Eaton JW (2020). Perceptions and behavioural responses of the general public during the COVID-19 pandemic: a cross-sectional survey of UK adults.

[53] Farber S, Johnson J. New data shows young people need to take social distancing seriously: Younger people may be spreading the virus among themselves. ABC News; 2020. https://abcnews.go.com/Health/data-shows-young-people-socialdistancing/story?id=71283384.

[54] Pfefferbaum B, North CS (2020). Mental health and the Covid-19 pandemic. N Engl J Med.

[55] Cullen W, Gulati G, Kelly BD (2020). Mental health in the COVID-19 pandemic. QJM An Int J Med.

[56] Betsch C, Schmid P, Heinemeier D, Korn L, Holtmann C, Böhm R (2018). Beyond confidence: development of a measure assessing the 5C psychological antecedents of vaccination. PLoS One.

[57] Kwok KO, Li KK, Wei WI, Tang A, Wong SYS, Lee SS (2021). Editor’s Choice: Influenza vaccine uptake, COVID-19 vaccination intention and vaccine hesitancy among nurses: a survey. Int J Nurs Stud.

[58] Williams SN, Armitage CJ, Tampe T, Dienes K (2020). Public perceptions and experiences of social distancing and social isolation during the COVID-19 pandemic: a UK-based focus group study. BMJ Open Open: BMJ Publishing Group.

[59] Kim Y, Yoon T, Sohn A (2021). Effects of COVID-19 knowledge, risk perception, subjective norms, and perceived behavioral control on preventive action intentions and preventive action practices in college students. kjhep.

[60] Jang S, Sohn A (2020). Understanding public perception of COVID-19 and preventive behaviors based on a semantic network analysis. kjhep.

[61] Dryhurst S, Schneider CR, Kerr J, Freeman ALJ, Recchia G, Van Der Bles AM (2020). Risk perceptions of COVID-19 around the world. J Risk Res.

[62] Li X, Liu Q (2020). Social media use, ehealth literacy, disease knowledge, and preventive behaviors in the COVID-19 pandemic: cross-sectional study on Chinese netizens. J Med Internet Res.

[63] Yodmai K, Pechrapa K, Kittipichai W, Charupoonpol P, Suksatan W (2021). Factors associated with good COVID-19 preventive behaviors among older adults in urban communities in Thailand. J Prim Care Community Health.

